# Outdoor secondhand smoke exposure in public places frequented by minors in the urban area of Hangzhou City, China: A cross-sectional study

**DOI:** 10.18332/tid/192129

**Published:** 2024-09-05

**Authors:** Yuhuan Sun, Dahui Wang, Yang Yi, Hongkun Chen, Yuchen Zhou, Geyao Huang, Falin Zhao

**Affiliations:** 1 School of Public Health, Hangzhou Normal University, Hangzhou, China

**Keywords:** secondhand smoke exposure, minors, cross-sectional study, on-site observation, China

## Abstract

**INTRODUCTION:**

Hangzhou Public Places Smoking Control Regulations (2019) have been gradually adopted, which explicitly stipulate that smoking is strictly prohibited in the outdoor areas of educational and healthcare institutions for minors. However, there are few studies reporting the exposure to secondhand smoke (SHS) in outdoor public places for minors in the urban area of Hangzhou City.

**METHODS:**

We aimed to assess the exposure to SHS in public spaces frequented by minors using on-site observations and questionnaires. In this cross-sectional study, the area was divided into core and non-core areas based on the spatial distribution and development process of the city. The core areas included the West Lake commercial district, extending to the Qiantang River, while non-core areas were located beyond this radius. Using stratified random sampling, 30 public places in each area were selected as observation sites. On-site observations measured SHS exposure and smoking control, and questionnaires were administered to 6 individuals at each site. The results were compared between the two investigation methods.

**RESULTS:**

Among the 57 valid observation points, 24.6% (14/57) did not display any no-smoking signs. Outdoor SHS exposure rate from on-site observation P1 (observing someone smoking or smelling tobacco smoke), on-site observation P2 (observing someone smoking or smelling tobacco smoke or seeing cigarette butts) and questionnaire survey P3, were 59.6% (95% CI: 45.7–72.2), 91.1% (95% CI: 79.7–96.7) and 41.0% (95% CI: 35.5–46.7), respectively.

**CONCLUSIONS:**

The outdoor SHS exposure in areas frequented by minors in the urban district of Hangzhou City remains high, coupled with a lack of awareness of SHS risks among underage individuals. Therefore, controlling outdoor SHS exposure in these key areas is a critical public health issue in Hangzhou, requiring further tobacco control efforts. On-site observation is an important and supplementary research method to investigate outdoor SHS exposure, especially to describe the SHS exposure of focus areas.

## INTRODUCTION

It has been two decades since the signing of the World Health Organization Framework Convention on Tobacco Control in 2003^1^, yet tobacco use remains a pervasive global issue with significant public health ramifications. Secondhand smoke (SHS) is known to contain over 4000 harmful chemicals, including tar, ammonia, nicotine, suspended particles, PM2.5, and more than a dozen carcinogens such as polonium-210. The Report on Smoking Hazards to Health in China 2020^2^ highlighted that exposure to SHS is associated with an increased risk of lung cancer, coronary heart disease, childhood asthma, and suggested links to nasal sinus cancer, rectal cancer, breast cancer, stroke, atherosclerosis, and higher risk of diabetes. Previous studies have demonstrated that exposure to SHS poses greater physiological risks to minors, including increased risks of AD dementia and stroke^3^, as well as heightened mortality from coronary heart disease^4^ and pancreatic cancer in adulthood^5^. Additionally, SHS exposure is associated with elevated diastolic blood pressure^6^, greater carotid media thickness (cIMT)^7^ and earlier onset of myopia^8^. For minors, exposure to SHS poses risks to mental health, including a higher incidence of depressive symptoms^9^, mental illness, behavioral problems, and cognitive impairments^10^. Additional studies have shown that childhood SHS exposure adversely affects rhinoconjunctivitis symptoms in both adolescents with and without asthma^11^, and is associated with mild sleep-disordered breathing symptoms and academic performance among US youth, in a dose-dependent manner^12-14^. There is no safe level of SHS exposure, and comprehensive smoking bans represent the sole effective strategy for mitigating SHS hazards.

By February 2021, over 20 cities in China, including Beijing, Shanghai, Shenzhen, and Qingdao, had implemented legislative measures. On 27 November 2009, Hangzhou City enacted the Hangzhou Public Places Smoking Control Regulations, hereafter referred to as the Regulations, which took effect on 1 March 2010, marking the introduction of smoke-free indoor public spaces in Hangzhou. With the implementation of these regulations and increased public awareness of tobacco hazards, compliance with smoking bans has improved. In 2019, Hangzhou introduced an updated version of the Regulations on the Prohibition of Smoking in Public Places, expanding the scope of smoking bans, enhancing penalties for non-compliance, and specifically prohibiting smoking in six types of outdoor public areas, three of which cater to minors^15^. These include educational and healthcare institutions focused on women and children such as nurseries, kindergartens, primary and secondary schools, children’s palaces, and children’s hospitals. This revision underscores Hangzhou’s enhanced tobacco control policies and its commitment to protecting vulnerable populations, especially minors. Concurrently, numerous domestic and international studies have examined outdoor SHS exposure among minors in various regions. In 2019, the prevalence of SHS exposure in outdoor public places among middle school students in Beijing was 60.3%^16^ and the youth SHS exposure rate was 64.1% in Tianjin^17^. Data from a nationally representative sample in the United States indicate that up to 38.1% of children aged 3–11 years and 32.1% of adolescents aged 12–17 years are exposed to SHS^18^. In a study based on 12 European countries, it was found that more than half of non-smokers reported SHS presence in the schools (52.0%) and hospitals (67.3%)^19^. Moreover, a study conducted in 11 European countries revealed that nicotine was detected in 45.9% of outdoor entrances to primary schools^20^. The prevalence of SHS exposure among minors in public places was higher than in indoor public places such as healthcare facilities (24.4%) and primary and secondary schools (23.4%), as reported in the 2018 China Adult Tobacco Survey^21^. The current evidence suggests that exposure to SHS represents a significant public health concern, both within domestic settings and in international contexts. Recognizing the increased vulnerability of children to SHS, 25 countries have implemented legislation prohibiting smoking in vehicles when individuals under 18 years of age are present. Furthermore, to safeguard children in outdoor settings, 60 nations have expanded these protections with bans on smoking in areas designated for children’s activities, such as playgrounds^22^. Hence, there is a crucial need to prioritize SHS prevention and control in settings frequented by minors, despite limited existing research in this domain. This study aims to investigate the current status of SHS exposure in these critical areas. Based on our findings, we intend to propose measures that facilitate compliance with the Regulations, thereby reducing SHS exposure and minimizing tobacco-related harm among minors.

## METHODS

### Study design

In this cross-sectional study, a questionnaire was employed in accordance with the methodology set forth by the World Health Organization for the investigation of SHS, and on-site observations were conducted in outdoor public places frequented by minors.

*Questionnaire survey*

A survey questionnaire was designed based on the 2018 Chinese Adult Tobacco Survey and relevant literature^21^ to align with the study’s objectives. The questionnaire targeted individuals aged 15–75 years (born between April 2006 and April 1946) intercepted at designated observation points. Each point was staffed with two trained surveyors who conducted individual interviews. The survey covered demographic variables (gender, age, education level, occupation, marital status, presence of children, household registration, average monthly income per capita), outdoor SHS exposure in public places frequented by minors and SHS-related knowledge and beliefs. Knowledge included the perceived risks associated with SHS in relation to the three diseases (adult heart disease, childhood respiratory diseases and adult lung cancer) and beliefs referring to attitudes towards SHS (‘What would you do when someone smokes in public places?’, and ‘If someone smokes next to you, would you move away automatically?’)

*On-site observation*

During May 2021, two investigators conducted on-site observations at various locations. Observations at medical facilities occurred on weekends from 8:00 to 17:00, aligning with peak service times for women and children. At educational institutions, observations took place Monday to Friday from 15:00 to 17:30, immediately following school dismissal. Each observation point featured two zones (depicted in Figure 2), extending 10 m outward from extension lines on both sides of the main entrance. Each zone was be observed for 20 minutes. Assessment criteria include the presence of no-smoking signs, availability of smoking facilities, display of tobacco advertisements, and presence of individuals discouraging smoking. Additionally, observations documented the presence of cigarette smoke, number of smokers, and quantity of discarded cigarette butts.

### Selection of observation points

Previous studies have highlighted substantial urban-rural disparities in SHS exposure, underscoring its close association with socio-economic status^23,24^. Hangzhou has experienced rapid urbanization in recent years, marked by multiple administrative expansions and adjustments. To better capture socio-economic variations, our study employed a stratified sampling approach based on spatial proximity rather than traditional administrative divisions^25^.

Over the past two decades, Hangzhou’s urban development has evolved from a ‘West Lake-centric’ phase to a ‘Qiantang River development’ phase. The West Lake area, characterized as an ‘old city’, boasts predominantly local residents with a robust history of infrastructure and economic development. Conversely, the broader city is characterized as a ‘new city’, characterized by a higher proportion of non-local residents and relatively shorter urban planning and economic development histories.

**Figure 1:**
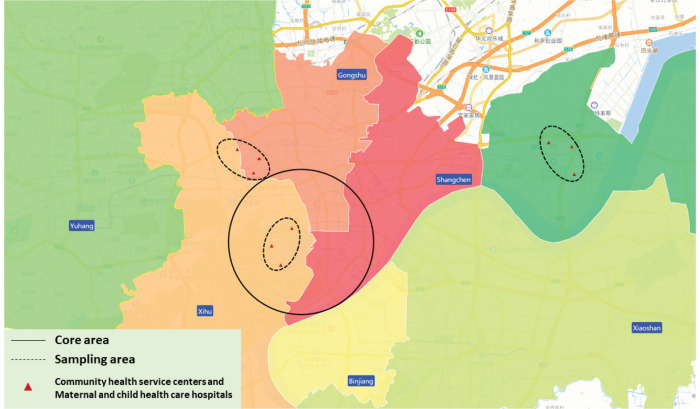
Schematic diagram of a stratified random sampling scheme based on spatial distribution, Hangzhou, 2021

Given these dynamics, our study utilized spatially stratified random sampling, focusing primarily on the West Lake commercial district. Initially, the urban area was categorized into core and non-core zones based on proximity to the city center. The core zone encompassed the West Lake commercial district, extending outward in a radius to the Qiantang River, while the non-core areas lay beyond this radius (Figure 1). The second stage involved forming combinations of community health service centers. Sample combinations were created by grouping 2–3 closely located community health service centers. A total of 30 sample combinations were randomly selected using a random number table method, encompassing 88 community health service centers and maternal and child healthcare hospitals in Hangzhou. From both core and non-core areas, five combinations were randomly chosen, resulting in 10 combinations comprising 30 community health service centers or maternal and child healthcare hospitals.

**Figure 2:**
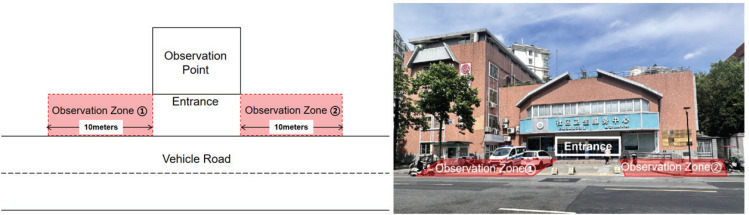
The schematic diagram outlining the delineation of the observation scope at each observation points, Hangzhou, 2021

The third stage consisted of selecting educational institutions near the medical facilities. Within each combination, one community health service center was randomly selected, alongside one kindergarten, one primary school, and one middle school in its vicinity. This totaled 30 educational institutions across their respective combinations. Ultimately, there were 10 combinations, each comprising three community health service centers, one kindergarten, one primary school, and one middle school, amounting to a total of 60 observation points.

### Sample size estimation

According to Jackson’s N:q rule, the recommended ratio of sample size to estimated parameter is 20:1, which may be relaxed to 10:1. Given a questionnaire comprising 19 items, the initial minimum sample size calculation yields 190. Accounting for potential biases such as invalid responses (estimated at 10%), the final sample size is determined by multiplying the number of items by 10 and adjusting by 1.1. Therefore, the minimum required sample size for this survey, with 19 items in the questionnaire, is 209.

### Definition of relevant concepts and indicators

*Key observation points*

Key observation points involving minors include educational institutions (kindergartens, primary and secondary schools, youth activity center, and educational training institutions), as well as healthcare institutions (maternal and child healthcare hospitals, children’s hospitals, and community health service centers).

*Secondhand smoke (SHS)*

SHS refers to the tobacco smoke released into the environment during combustion, comprising both exhaled smoke from smokers and emissions from burning tobacco.

*SHS exposure assessment*

SHS exposure is assessed using three indicators defined as follows:

- P_1_: On-site observation involves identifying individuals smoking or detecting the odor of smoke at specified observation points.
- P_2_: On-site observation includes identifying individuals smoking, detecting the odor of smoke, or noting the presence of cigarette butts in the observed area.

Perceived SHS exposure:

- P_3_: In the questionnaire survey, this refers to witnessing outdoor smoking in key areas for minors within the past 30 days, involving both smokers and non-smokers.

*Risks associated with SHS exposure*

Understanding the risks associated with SHS exposure includes awareness that SHS can contribute to adult lung cancer, childhood respiratory diseases, and adult heart disease.

*Regional differentiation in observation points*

Regional differentiation in observation points: using the central area of the West Lake Business District in Hangzhou as the focal point, we defined a circular boundary with the distance from this central area to the Qiantang River as the radius. The area within the circle represents the core area, while the area outside constitutes the non-core area.

### Statistical analysis

The double-entry method was used to input investigator-recorded data into Epidata software. Descriptive statistics were initially computed for all variables. Exposure rates and their 95% confidence intervals (CIs) were used to represent the exposure to SHS in different areas and different observation methods, non-overlapping confidence intervals indicated statistical differences. In the questionnaire survey section, the comparisons of demographic characteristics between the core area and the non-core area were conducted using the chi-squared test. The difference in SHS exposure between core and non-core areas was compared by chi-squared test and logistic regression model; the model was adjusted for potential confounding factors in three different models. All tests were two tailed and p<0.05 was considered statistically significant. Statistical analyses were performed using SPSS (version 26.0).

## RESULTS

### Questionnaire survey

A total of 360 questionnaires were distributed in this survey, excluding those with missing values, resulting in 312 valid responses and an effective response rate of 86.7%. Table 1 presents demographic information of Hangzhou residents aged 15–75 years, across various regions. Residents in the core area, compared to those in non-core areas, generally had an older age, higher average monthly household income, and were predominantly engaged in mental labor.

**Table 1:** Demographic characteristics of survey participants in different areas, Hangzhou, 2021 (N=312)

*Characteristics*	*Total n (%)*	*Core area n (%)*	*Non-core area n (%)*	*χ^2^*	*p*
**Gender**					
Male	141 (45.2)	65 (46.1)	76 (53.9)	0.050	0.823
Female	171 (54.8)	81 (47.4)	90 (52.6)		
**Age** (years)					
15–30	64 (20.5)	31 (48.4)	33 (51.6)	16.196	0.001
31–40	105 (33.7)	35 (33.3)	70 (66.7)		
41–55	71 (22.8)	34 (47.9)	37 (52.1)		
56–75	72 (23.1)	46 (63.9)	26 (36.1)		
**Household registration**					
Urban	207 (66.3)	101 (48.8)	106 (51.2)	0.986	0.321
Rural	105 (33.7)	45 (42.9)	60 (57.1)		
**Education level**					
Junior high school or lower	85 (27.2)	39 (45.9)	46 (54.1)	0.341	0.843
Senior high school/vocational school	59 (18.9)	26 (44.1)	33 (55.9)		
College degree or higher	168 (53.8)	81 (48.2)	87 (51.8)		
**Marital status**					
Married	251 (80.4)	118 (47.0)	133 (53.0)	0.024	0.876
Unmarried	61 (19.6)	28 (45.9)	33 (54.1)		
**Parental status**					
Yes	250 (80.1)	117 (46.8)	133 (53.2)	0.000	0.997
No	62 (19.9)	29 (46.8)	33 (53.2)		
**Average monthly household income** (CNY)					
<5000	90 (29.7)	36 (40.0)	54 (60.0)	9.342	0.009
5000–10000	119 (39.3)	49 (41.2)	70 (58.8)		
>10000	94 (31.0)	56 (59.6)	38 (40.4)		
**Occupational nature**					
Mentally demanding	148 (48.4)	70 (47.3)	78 (52.7)	25.329	<0.001
Physically demanding	100 (32.7)	31 (31.0)	69 (69.0)		
Other	58 (19.0)	42 (72.4)	16 (27.6)		

CNY: 1000 Chinese Yuan about US$140.

In critical contexts concerning minors, the overall rate of outdoor SHS exposure was 41.0%. There was no statistically significant difference in SHS exposure rates between educational settings and healthcare settings (38.0%; 95% CI: 26.5–50.9 vs 44.7%; 95% CI: 32.4–57.7). However, outdoor SHS exposure in the core area was higher compared to the non-core area (56.8%; 95% CI: 48.3–64.8 vs 27.1%; 95% CI: 20.6–34.6) (Table 2).

**Table 2:** Outdoor SHS exposure among minors in different areas and types of key outdoor public areas, Hangzhou, 2021 (N=312)

	*Total n*	*Exposure to SHS n (%)*	*No exposure to SHS n (%)*	*χ^2^*	*p*
**Region**					
Core	146	83 (56.8)	63 (43.2)	28.399	<0.001
Non-core	166	45 (27.1)	121 (72.9)		
**Premises**					
Educational institutions	171	65 (38.0)	106 (62.0)	1.421	0.233
Healthcare Institutions	141	63 (44.7)	78 (55.3)		
**Total**	312	128 (41.0)	184 (59.0)		

The core and non-core areas were divided based on the spatial distribution and development process of the city. The core areas included the West Lake commercial district, extending to the Qiantang River, while non-core areas were located beyond this radius.

Demographic data were adjusted by logistic regression analyses which showed that there were still differences in SHS exposure between core and non-core areas (AOR=3.883; 95% CI: 2.317–6.341), p<0.001) (Table 3).

**Table 3:** Comparison of the SHS exposure rates between the core and non-core areas using logistic regression model, Hangzhou, 2021 (N=312)

*Variable*	*Model 1*	*Model 2*	*Model 3*
	*OR (95% CI)*	*AOR (95% CI)*	*AOR (95% CI)*
Core vs Non-core (ref.)	3.543 (2.206–5.689)*	4.130 (2.388–7.144)*	3.883 (2.317–6.341)*

AOR: adjusted odds ratio. Model 1: unadjusted. Model 2: adjusted for age, average monthly household income and occupational nature. Model 3: adjusted as for Model 2 plus gender, household registration, education level, marital status and parental status.

* p<0.001.

Regarding awareness of the hazards of outdoor SHS, 81% of respondents had acquired knowledge through various channels. Among them, 88.7% (95% CI: 84.4–92.0) recognize that SHS can cause lung cancer in adults, while only 31.5% (95% CI: 23.0–41.3) are aware of its links to respiratory diseases in children and heart disease in adults. Furthermore, 78.8% of survey participants were familiar with laws, regulations, or relevant provisions regarding smoking bans in public places. The survey also reveals that when encountering someone smoking in public areas, only 11.9% of individuals would intervene, 80.0% express aversion without interference, and 83.9% would simply move away.

### On-site observation

**Figure 3:**
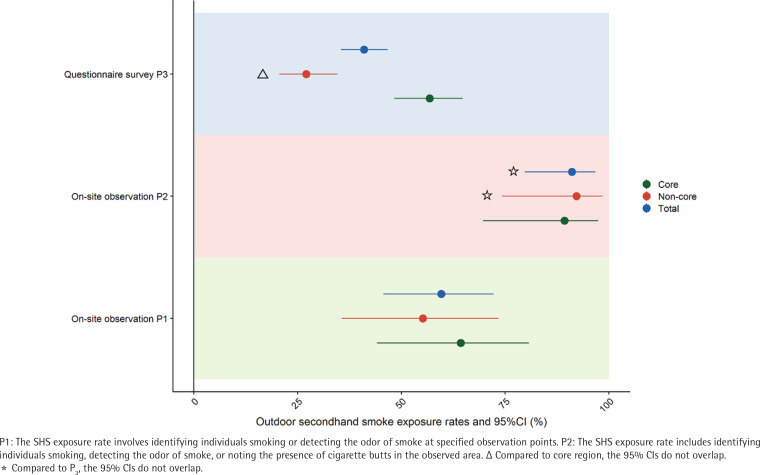
Comparison of outdoor SHS exposure rates by different areas and survey methods, Hangzhou, 2021

After excluding observations with missing values, 59.6% of the 57 valid observations showed exposure to outdoor SHS and 57.1% were smoking. Table 4 presents various indicators of SHS exposure across different areas. SHS exposure rates did not significantly differ between educational settings and healthcare settings (57.1%; 95% CI: 31.2–79.9 vs 62.1%; 95% CI: 37.0–82.5), nor between core and non-core areas (64.3%; 95% CI: 44.1–80.7 vs 55.2%; 95% CI: 35.7–73.4). Interestingly, when SHS exposure was defined by multiple indicators such as detecting smoke smell or observing cigarette butts, the exposure rate rose to 91.1% (Table 4 and Figure 3). Furthermore, nearly 25% of observation points lacked no-smoking signs, and 17.5% had improperly installed smoking devices. None of the observation points displayed tobacco advertisements, yet no actions were taken to discourage smoking.

**Table 4:** The different levels of outdoor SHS exposure in different regions as indicated by different survey methods, Hangzhou, 2021

*Region*	*On-site observation*		*Questionnaire survey*
	*P1 % (95% CI)*	*P2 % (95% CI)*	*P3 % (95% CI)*
Core	64.3 (44.1–80.7)	89.3 (69.7–97.4)*	56.8 (48.3–64.8)
Non-core	55.2 (35.7–73.4)*	92.2 (74.2–98.5)*	27.1 (20.6–34.6)^§^
Total	59.6 (45.7–72.2)	91.1 (79.7–96.7)*	41.0 (35.5–46.7)

P1: On-site observation entails observing individuals smoking or detecting the smell of smoke at the designated observation points. P2: On-site observation entails observing individuals smoking, detecting the smell of smoke, or observing the presence of cigarette butts in the observed area. P3: In the questionnaire survey, witnessing outdoor smoking in key areas for minors within the past 30 days, involving both smokers and non-smokers.

* Indicates statistically significant difference compared to P3.

§ Indicates statistically significant difference compared to core region.

## DISCUSSION

Our survey indicates that awareness regarding the respiratory risks of SHS in children stands at only 31.5%, markedly lower than the 71.4% reported in the 2018 China Adult Tobacco Survey^21^. This underscores the need for intensified tobacco control advocacy to highlight the substantial health hazards SHS poses to minors. Only a few people will actively try to stop others from smoking, and the vast majority will choose to ignore the problem. It is therefore necessary to ensure individuals are fully informed of their rights to refuse and prevent exposure to secondhand smoke through various public awareness campaigns. On the other hand, nearly a quarter of establishments are non-compliant with regulations due to a lack of required no-smoking signs. These signs, which should include graphic warnings against smoking, information on legal responsibilities for smoking violations, and contact details for reporting complaints, are absent. Additionally, there is a lack of intervention or discouragement of smoking in these venues. Moreover, almost one-fifth of establishments provide smoking facilities, potentially leading smokers to inadvertently smoke in non-smoking areas. This contradicts regulations stating that smoking accessories must not be present in areas where smoking is prohibited. As previously stated by Li et al.^26^, the main barriers to smokers’ compliance with smoking bans in public spaces at the organizational level were the ineffective implementation of these bans. Following the implementation of the Framework Convention on Tobacco Control (FCTC) in a number of countries, it was determined that the more rigorous the enforcement of the Convention, the more effective the control of tobacco use^1^. Therefore, strengthening supervisory and management efforts by health authorities is crucial to ensuring compliance among operators and managers of smoke-free and smoking-restricted areas, thereby fulfilling their responsibilities under smoking bans. It is also important to involve relevant stakeholders, such as government departments such as the Ministry of Health, regulatory agencies and the Ministry of Education, medical associations such as pediatricians, and organized civil society groups such as children’s rights advocates, in the implementation of a monitoring and surveillance system that complies with the law. And it is also important to carry out an extensive campaign for law enforcement through mainstream media or other effective social media platforms.

In the revised Regulations, specific key areas such as educational and healthcare institutions for minors were included in designated outdoor smoking-free zones in public spaces. Subsequently, we conducted the first survey on SHS exposure in these specified locations. These areas are crucial as they are where minors spend considerable time, and the environmental quality directly affects their physical and mental well-being^27,28^. Our findings substantiate that outdoor SHS exposure remains notably elevated in critical venues for minors across various areas of Hangzhou. The outdoor SHS exposure rates obtained from on-site observations (59.6%) and questionnaires (41.0%) in our study exceed those reported by Zhang et al.^29^ for indoor public places in hospitals (7.79%) and schools (11.36%) in Hangzhou, as well as the Jiang et al.^25^ rates for SHS exposure indoors in healthcare institutions (13.3%) and on primary and secondary school campuses (11.3%). Furthermore, children and adolescents exposed to smoking at school entrances encounter pro-smoking cues within their educational environment, potentially influencing their attitudes and beliefs regarding tobacco use^20,30,31^. Therefore, effectively preventing and controlling outdoor SHS exposure in critical areas for minors remains a paramount urban public health priority in Hangzhou.

In recent years, Hangzhou’s urban development has shifted from the West Lake area to the Qianjiang area, pivoting its focus from the West Lake to the Qiantang River. This transformation encompasses urban planning, economic development, transportation, and environmental protection. Significant milestones include the establishment of the Hangzhou Economic and Technological Development Zone, hosting the Asian Games, and the growth of numerous high-tech and internet industries. This study employed a division between core and non-core areas to ensure representative sample selection. The core area, comprising the older central urban zone of Hangzhou, exhibits higher economic activity and an older demographic. Residents tend to be older, with fewer physically demanding jobs and higher average incomes, aligning with area characteristics. The survey reveals higher outdoor SHS exposure in the core area compared to non-core areas, potentially linked to higher smoking rates among residents of advanced age.

Referring to the 2010 Global Adult Tobacco Survey China Report^32^ and the 2018 China Adult Tobacco Survey^21^, the definition of SHS exposure in public places is based on the proportion of individuals who reported witnessing smoking or smelling tobacco smoke among those who visited specific locations within the past 30 days. In Hangzhou’s urban areas, a study observed that within a 20-minute timeframe, 59.6% of key outdoor public areas frequented by minors (such as educational and healthcare institutions) met the criterion of ‘observing someone smoking or smelling tobacco smoke (P1)’. Including the criterion of ‘seeing cigarette butts on the ground (P2)’ raised this exposure rate to 91.1%. The indicators used in the on-site observations were similar to those used in several studies by Henderson et al.^20,33^, mainly including smell of smoke, people smoking and cigarette butts, suggesting that these indicators can serve as reliable scientific indices for the assessment of SHS. Additionally, questionnaire surveys indicated an overall SHS exposure rate of 41.0% within the past 30 days in these key outdoor public areas. Therefore, to accurately assess SHS exposure among residents in different areas, it is advisable to utilize multiple SHS indicators. For instance, the presence of discarded cigarette butts can indirectly indicate recent smoking activity in specific locations. SHS exposure rates obtained from questionnaires may underestimate actual exposure rates due to recall bias. It is crucial to comprehensively analyze findings from both questionnaire surveys and on-site observations. On-site observation serves as a valuable supplementary method to investigate outdoor SHS exposure, enabling a comprehensive collection of diverse indicators for a more precise assessment of SHS levels in targeted areas. This evaluation is essential for gauging the efficacy of new regulations and policies implemented across various locales.

### Strengths and limitations

Since the enactment of the revised Hangzhou Public Places Smoking Control Regulations in 2019, this study has pioneered research and analysis on SHS exposure in key venues frequented by minors, employing a novel approach combining questionnaire surveys and on-site observations. However, the study faces three primary limitations. Firstly, despite selecting nearly one-third of Hangzhou City’s community health service centers, the sample size remains small, resulting in wide confidence intervals for outdoor SHS exposure rates. Secondly, the questionnaire surveys utilized intercept sampling, a non-probability method that captures a representative cross-section of the sample area but lacks robust statistical inference capabilities. Thirdly, this study is a research analysis based on the tobacco control regulations in Hangzhou, China, which is more limited in terms of the importance of promotion at the regulatory level due to the fact that relevant regulations vary from country to country.

## CONCLUSIONS

Although smoking rates and exposure to secondhand smoke have declined since the implementation of the new version of the Hangzhou regulations^34^, outdoor SHS exposure remains notably high in specified areas frequented by minors within Hangzhou’s urban district. Awareness of the risks associated with SHS among minors is notably low. Addressing outdoor SHS exposure in these critical zones thus remains a significant public health concern in Hangzhou, necessitating enhanced tobacco control measures. Moreover, on-site observation proves crucial as a supplementary method to comprehensively assess outdoor SHS exposure, particularly in detailing exposure dynamics within focal areas^34,35^.

## Data Availability

Data might be available upon reasonable request to the authors.
